# Syntax Score and Major Adverse Cardiac Events in Patients with Suspected Coronary Artery Disease: Results from a Cohort Study in a University-Affiliated Hospital in Southern Brazil

**DOI:** 10.5935/abc.20160111

**Published:** 2016-09

**Authors:** Felipe C. Fuchs, Jorge P. Ribeiro, Flávio D. Fuchs, Marco V. Wainstein, Luis C. Bergoli, Rodrigo V. Wainstein, Vanessa Zen, Alessandra C. Kerkhoff, Leila B. Moreira, Sandra C. Fuchs

**Affiliations:** Hospital de Clínicas de Porto Alegre - Universidade Federal do Rio Grande do Sul, Porto Alegre, RS - Brazil

**Keywords:** Coronary Artery Disease / epidemiology, Probability, Cineangiography, Syntax Score, Cohort Studies

## Abstract

**Background:**

The importance of coronary anatomy in predicting cardiovascular events is well known. The use of traditional anatomical scores in routine angiography, however, has not been incorporated to clinical practice. SYNTAX score (SXscore) is a scoring system that estimates the anatomical extent of coronary artery disease (CAD). Its ability to predict outcomes based on a baseline diagnostic angiography has not been tested to date.

**Objective:**

To evaluate the performance of the SXscore in predicting major adverse cardiac events (MACE) in patients referred for diagnostic angiography.

**Methods:**

Prospective cohort of 895 patients with suspected CAD referred for elective diagnostic coronary angiography from 2008 to 2011, at a university-affiliated hospital in Brazil. They had their SXscores calculated and were stratified in three categories: no significant CAD (n = 495), SXscore_LOW-INTERMEDIATE_: < 23 (n = 346), and SXscore_HIGH_: ≥ 23 (n = 54). Primary outcome was a composite of cardiac death, myocardial infarction, and late revascularization. Secondary endpoints were the components of MACE and death from any cause.

**Results:**

On average, patients were followed up for 1.8 ± 1.4 years. The primary outcome occurred in 2.2%, 15.3%, and 20.4% in groups with no significant CAD, SXscore_LOW-INTERMEDIATE_, and SXscore_HIGH_, respectively (p < 0.001). All-cause death was significantly higher in the SXscore_HIGH_ compared with the 'no significant CAD' group, 16.7% and 3.8% (p < 0.001), respectively. After adjustment for confounding factors, all outcomes remained associated with the SXscore.

**Conclusions:**

SXscore independently predicts MACE in patients submitted to diagnostic coronary angiography. Its routine use in this setting could identify patients with worse prognosis.

## Introduction

The importance of coronary anatomy in predicting cardiovascular events has been known for decades, when studies like CASS (Coronary Artery Study) registry were published.^1^ This large cohort study showed the ability of anatomical scores of coronary artery disease (CAD) to predict events, but their routine use was not incorporated to clinical practice.^2^ Nowadays, standard of care indicates functional, noninvasive, assessment of ischemia, such as stress-echocardiogram, nuclear imaging and magnetic resonance imaging, to evaluate patients with known or suspected CAD.^3^ Nonetheless, a significant number of patients are eventually submitted to coronary angiography for diagnostic confirmation.^3^ Therefore, re-assessing the performance of anatomical scores to predict outcomes, in a context of newer clinical and interventional therapies, is potentially worthwhile. Currently, the SYNTAX (Synergy between percutaneous coronary intervention with Taxus and Cardiac Surgery) Score (SXscore), a more elaborate method to quantify anatomic lesions, is an available online tool that estimates the anatomical extent of CAD.^4^

The SXscore is a comprehensive angiographic scoring system based on coronary anatomy and lesion characteristics.^4^ It was initially developed to determine the extent of CAD and lesion complexity, which reflect the difficulties in performing myocardial revascularization, particularly percutaneous coronary interventions (PCI). In the SYNTAX trial, high SXscore values (above 33) identified patients in whom coronary artery bypass grafting (CABG) resulted in better outcomes than in patients submitted to percutaneous revascularization.^5^ Five-year follow-up of this trial identified patients with scores above 22 as more suitable for CABG.^6^

The SXscore was developed as a tool in the decision-making process and, later on, its usefulness was expanded as a predicting score of major adverse cardiac events (MACE) in patients submitted to PCI.^7-14^ Those studies included elective and urgent revascularization procedures. However, the majority of coronary angiographies are done for diagnostic purposes.^15^ The prognostic performance of the SXscore in that setting has not been reported to date, and is the aim of this investigation.

## Objective

To evaluate the performance of the SXscore in predicting MACE in patients referred for diagnostic angiography.

## Methods

### Study design and population

This cohort study enrolled patients with suspected CAD referred for elective, diagnostic coronary angiography, from 2008 to 2011, at a reference tertiary university-affiliated hospital (Hospital de Clínicas de Porto Alegre), in Southern Brazil. The patients were referred by cardiologists from the public health system and private practices, and underwent cardiac catheterization due to suspected CAD with or without previous noninvasive testing for ischemia. Patients referred for angiography due to suspected CAD and associated valvular heart disease were also included. Men and women aged 40 years or over were eligible for the study, excluding those with previous coronary revascularization (surgical or percutaneous), class III or IV heart failure, chronic renal disease (previous medical diagnosis or serum creatinine greater than 1.5 mg/dL), history of cancer, or severe psychiatric illness. Patients admitted to the hospital for acute coronary syndromes were not included.

### Enrollment and study procedures

The study protocol was approved by the hospital's Ethics Committee, which is accredited by the Office for Human Research Protections as an Institutional Review Board, and informed, written consent was obtained. Interviews pertaining demographic information, lifestyle characteristics, and past medical history were done using a standardized questionnaire. After the angiographies, the patients' attending physicians were responsible for assessing the need for revascularization and all medical treatment. The follow-up was conducted from 2008 to 2012.

### SYNTAX score and angiographic analysis

SXscores were calculated prospectively by scoring all coronary lesions producing a ≥ 50% diameter stenosis in vessels ≥ 1.5 mm, using the algorithm that is available at the SYNTAX score website.^16^ Subsequently, they were categorized as: SXscore_HIGH_ (≥ 23); SXscore_LOW-INTERMEDIATE_ (< 23); and no significant CAD (reference category). Two interventional cardiologists (FCF, LCCB) independently performed the angiographic visual analysis for the assessment of the score. They were trained in calculating the SXscore using the website tutorial. Afterwards, they scored another 80 cases, which were extensively discussed with senior interventional cardiologists. Inter- and intra-observer agreement for determination of the SXscore was evaluated in another group of 90 angiographies.

### Study endpoints

The primary endpoint was MACE, defined as a time to first event among cardiac death, myocardial infarction (MI) or late revascularization. Myocardial infarction and revascularization followed by death in the same hospitalization were adjudicated as cardiac deaths. Cardiac death was defined, additionally, as sudden death. Myocardial infarction was diagnosed by an increase or decrease of biomarkers, in the presence of symptoms, ECG abnormalities suggestive of ischemia.^17^ Some patients were treated for acute MI in other hospitals and the diagnosis was defined on the basis of the discharge diagnosis. Late revascularization was either PCI or CABG.

Percutaneous and surgical revascularizations based on diagnostic angiography findings, performed until three months after the angiography, were defined as index procedures and not considered outcomes. Interventions performed during follow-up, non-directly related to the diagnostic angiography, were defined as late revascularizations and included in the primary outcome. Secondary endpoints were cardiac death, cardiovascular death (fatal MI or stroke), MI, coronary revascularization, and overall mortality.

All deaths were confirmed through verbal autopsy,^18^ death certificate (obtained at the Government's Health Department, which has all state death records), or hospital records. Myocardial infarction was established by hospitalization, with diagnosis informed by a physician. An independent Clinical Events Committee adjudicated all endpoints. Data collection regarding the outcomes underwent control of quality to verify reliability, and another investigator checked 5% of the verbal autopsies.

### Sample size calculation and statistical analysis

The questionnaires were coded and entered into a database using Epinfo 2004 software (version 3.3.2, Centers for Disease Control and Prevention, Atlanta, USA), with data entry quality control to verify amplitude and consistency. A sample size of 588 participants would be necessary to identify a hazard ratio (HR) of at least 2.4, with 80% power and 5% significance level (two-tailed), considering that 5% of unexposed and 12% of exposed to the highest score, a 1:1.5 ratio, respectively, would present a primary endpoint. Considering the lack of previous reports about the performance of SXscore to predict events in this context, sample size was increased to 906 participants to include enough patients with high scores (>23) to provide adequate statistical power. Epi Info 2004, Statcalc module, was used for sample size calculation.

Inter and intra-observer reliability was assessed by crosschecking 90 angiograms evaluated by two interventional cardiologists and reviewed by a third one. Agreement using Kappa coefficient was done, and interpreted according to Feiss et al.^19^ Substantial agreement was defined by a Kappa coefficient of 0.7, considering the proportion of patients with SXscore > 23, being 30% according to observer 1 and 20% by observer 2, with accuracy of 0.2. Intraclass correlation coefficients were also calculated.

Recommendations of the STARD^20^ were used to plan and report this study. Data are presented by mean ±SD, percentages and HR with 95% confidence intervals (CI). Receiver operating characteristic (ROC) curve was used to calculate C-statistic and the area under the curve. Kaplan-Meier survival curve for MACE was calculated for patients according to SXscores. Multivariate analysis of the predictive power of the SXscore was performed using Cox regression, which allowed the estimation of HR and 95% CI. Variables associated to the outcome in the bivariate analysis (P ≤ 0.2) were eligible as confounding factors. Considering that many variables are intermediates in the causation of MACE, they were individually evaluated to be included or not in the analysis. The same model was run having the number of diseased vessels (none, one and multivessel) as exposure variable. The analyses were carried out using the Statistical Package for Social Sciences (SPSS®, version 17, Chicago, IL, USA) software, and a p value <0.05 was regarded as statistically significant.

## Results

**Figure 1:**
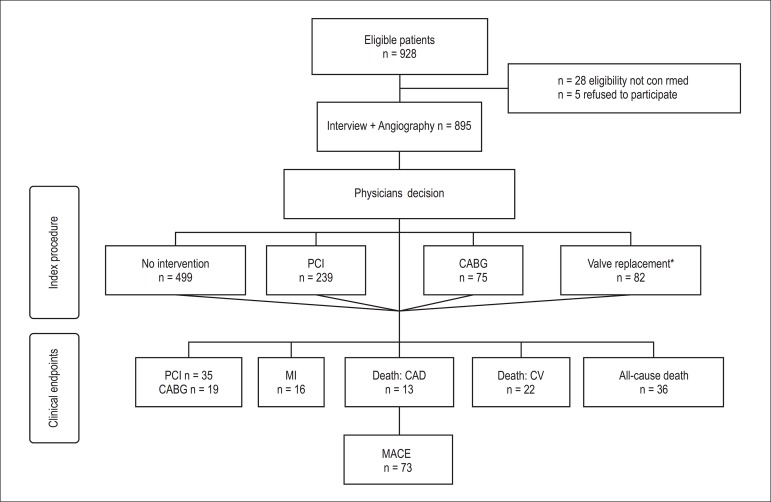
Flowchart of patients. PCI: percutaneous coronary intervention; CABG: coronary artery bypass grafting; MI: myocardial infarction; CAD: coronary artery disease; CV: cardiovascular; MACE: major adverse cardiac events. * Valve replacement patients were excluded from the outcome analysis. ^Ŧ^ MACE as defined in methods section.

Study flowchart is presented in Figure 1. Among 928 eligible patients, 895 patients with SXscore were included in the cohort and were followed up on average for 1.8 ± 1.4 years. After angiography, 314 (35.1%) patients were submitted to PCI or CABG, and 82 (9.2%) to valve replacement (index procedure). New interventions were done during the follow-up (late revascularizations) in 54 patients (35 percutaneous and 19 surgical). Myocardial infarction occurred in 16 patients, cardiac death in 13, cardiovascular death in 22, and all-cause death in 40 patients. MACE was established in 73 patients.

Spearman coefficient between the SXscores calculated by the two interventional cardiologists was 0.902 (p < 0.001), and the interobserver agreement between them was 0.94 (95% CI: 0.91-0.96). Kappa coefficient was 0.83 for the two interventional cardiologists. There were 495 patients with a score of 0 (55.4%) and 400 (44.6%) with positive scores, ranging from 1 to 43, with a mean of 12.6 (95% CI: 11.7-13.4). Patients with coronary lesions ≥ 50% diameter stenosis in vessels ≥1.5 mm were classified as SXscore_LOW-INTERMEDIATE_ (n = 345) or SXscore_HIGH_ (n = 54).

Baseline clinical and angiographic characteristics according to patient categories are presented in Table 1. The mean age of patients with SXscore_HIGH_ was higher than that of patients with SXscore_LOW-INTERMEDIATE_. The proportion of men, patients with diabetes mellitus and hypertension was higher among patients with SXscore_HIGH_ as well. Clinical indications for diagnostic coronary angiography were not remarkably different by the SXscore, but more patients in the SXscore_LOW-INTERMEDIATE_ had typical CAD symptoms and more patients without significant CAD had other symptoms. As expected, prevalence of multivessel disease and all markers of increased lesion complexity, such as the presence of total occlusions, bifurcations and small vessel disease, were significantly more frequent in the SXscore_HIGH_ category.

**Table 1:** Baseline clinical characteristics

Baseline characteristics	No significant CAD n = 495	SXscore_LOW-INTERMEDIATE_ n = 346	SXscore_HIGH_ n = 54	p value
Age	59.1 ± 10.4	60.8 ± 9.6	63.6 ± 8.6	0.002
Male	234 (47.3)	226 (65.3)	39 (72.2)	< 0.001
Diabetes mellitus	92 (18.6)	85 (24.6)	18 (33.3)	0.01
Current smoking	65 (13.2)	51 (15.0)	3 (5.6)	0.08
Hypertension	344 (69.8)	260 (76.5)	45 (83.3)	0.02
Symptoms of CAD only	128 (25.9)	115 (33.4)	14 (25.9)	0.05
With a positive noninvasive test	209 (42.2)	139 (40.2)	28 (51.9)	0.3
Valve disease with suspected CAD	14 (2.8)	3 (0.9)	1 (1.9)	0.14
Other complaints	46 (9.3)	13 (3.8)	1 (1.9)	0.002
Angiographic analysis*				
Right dominance	-	309 (89.3)	51 (94.4)	0.3
Nº. lesions per patient	-	1.8 ± 1.0	3.9 ± 1.5	< 0.001
Total occlusions	-	88 (25.4)	41 (75.9)	< 0.001
Bifurcations	-	125 (36.1)	44 (81.5)	< 0.001
Small vessels /diffuse disease	-	68 (19.7)	26 (48.1)	< 0.001
Left main	-	13 (13.8)	13 (24.1)	< 0.001
Left anterior descending	-	218 (63.0)	47 (87.0)	< 0.001
Left circumflex	-	109 (31.5)	36 (66.7)	< 0.001
Right coronary artery	-	169 (48.8)	43 (79.6)	< 0.001
One vessel disease	-	196 (56.7)	4 (7.4)	< 0.001
Multivessel disease or LM	-	150 (43.3)	50 (92.6)	< 0.001

Values are given as n (%) or mean ±SD. LM: left main; CAD: coronary artery disease.

* Assessment using the SYNTAX score definitions.

### Procedures after index angiography

The proportion of patients submitted to PCI, CABG and valve replacement based on the index diagnostic angiography according to patient category is presented in Table 2. As expected, more patients with higher scores were submitted to CABG. Despite having no significant CAD based on angiographic assessment done for this study, 3.4% of patients were submitted to PCI.

**Table 2:** Treatment after the index angiography and cumulative clinical outcomes across patient categories

Type of procedure	No significant CAD n = 495	SXscore_LOW-INTERMEDIATE_ n = 346	SXscore_HIGH_ n = 54	p value
**Interventions:**				< 0.001
Percutaneous Coronary Intervention	17 (3.4)	208 (60.3)	14 (25.9)	
Coronary Artery Bypass Surgery	0	46 (13.3)	29 (53.7)	
Isolated valve replacement*	78 (15.8)	4 (1.2)	0	
No invasive intervention	400 (80.8)	88 (25.4)	11 (20.4)	
All-cause death	16 (3.9)	11 (3.3)	9 (16.7)	< 0.001
Cardiac death and MI	4 (1.0)	13 (3.9)	9 (16.7)	< 0.001
Cardiovascular death	9 (2.2)	6 (1.8)	7 (13)	< 0.001
MACE	9 (2.2)	53 (15.7)	11 (20.4)	< 0.001

Values are given as n (%).

* patients excluded from outcome analysis. MI: myocardial infarction; MACE: MI, cardiac death, and late revascularization.

### Clinical outcomes

**Figure 2:**
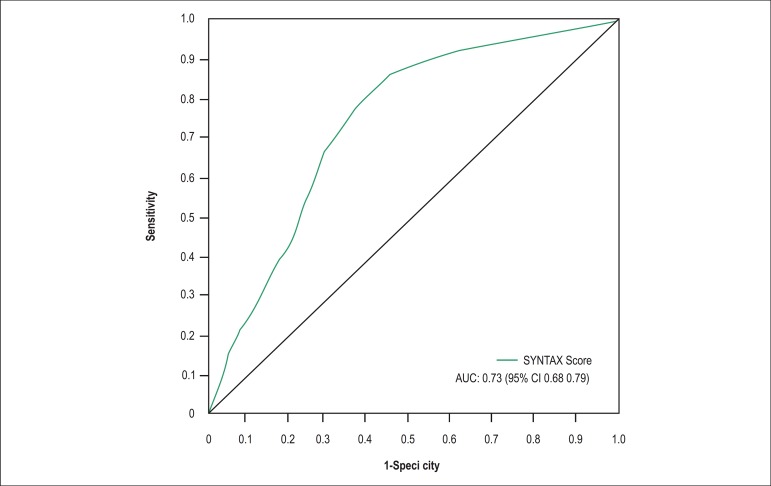
ROC curve for the SYNTAX score.

The cumulative incidence of clinical outcomes across patient groups is shown in Table 2. All-cause death was significantly higher in SXscore_HIGH_ patients as compared with patients without significant CAD, 16.7% and 3.9% (p < 0.001), respectively. Cardiovascular death, non-fatal MI, and late revascularization were more frequent in the SXscore_HIGH_ as well. After adjustment for confounding factors, all outcomes remained associated with the SXscore (Table 3). Risk ratios for MACE, cardiac death or non-fatal MI and non-fatal MI alone were significantly associated with SXscore_LOW-INTERMEDIATE_ as well. Patients in the SXscore_HIGH_ category had a 12.5 (95% CI: 5.1-30.6) higher chance of presenting the primary outcome than those without significant CAD. This finding was similar among men (10.1; 95% CI: 3.9-25.9) and women (11.5; 95% CI: 1.1-117.3). Further adjustment for index revascularization did not change the estimates significantly. After adjustment for confounding factors, the primary outcome was also associated with the SXscore as a continuous variable (HR 1.06; 95% CI: 1.04-1.08). The area under the ROC curve was 0.73 (95% CI: 0.68-0.79) (Figure 2).

**Table 3:** Hazard ratios* for major clinical outcomes according to patient categories

Type of Event	No significant CAD† n = 495	SXscore_LOW-INTERMEDIATE_ n = 346	SXscore_HIGH_ n = 54	p value
All-cause death	1.0	0.8 (0.4-1.7)	4.3 (1.8-10.1)	< 0.001
Cardiovascular death	1.0	0.7 (0.3-2.1)	5.7 (2.0-15.9)	< 0.001
Cardiac death	1.0	1.3 (0.3-5.9)	11.8 (2.9-48.5)	< 0.001
MACE‡	1.0	7.2 (3.5-14.7)	12.5 (5.1-30.6)	< 0.001
Cardiac death or MI	1.0	3.5 (1.1-10.8)	16.0 (4.9-52.9)	< 0.001
MI	1.0	12.6 (1.6-98.3)	33.9 (3.7-308.0)	0.007
Late Revascularization	1.0	9.9 (4.2-23.4)	4.0 (0.8-20.0)	< 0.001

MI: myocardial infarction.

* Adjusted for age, sex and diabetes.

† Reference category,

‡ MACE: MI, cardiac death, and late revascularization.

**Figure 3:**
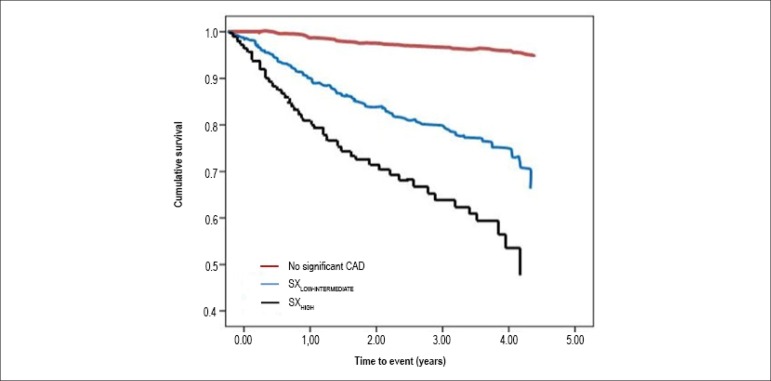
Kaplan-Meier Curve for MACE according to patient category. CAD: coronary artery disease.

MACE-free survival curves for patients according to SXscores are presented in Figure 3, showing that curves diverged immediately after angiography and further during follow-up. Risk ratios for MACE according to the number of diseased vessels, compared to none, were 6.9 (95% CI: 3.4-13.9) for one-vessel disease and 10.2 (5.2-20.1) for multivessel disease. Despite the intrinsic relationship of this classification and the SXscore, 42.0% of the patients with multivessel disease were classified in the SXscore_LOW-INTERMEDIATE_ category.

## Discussion

This study demonstrated that, in patients submitted to diagnostic angiography for suspected CAD, the SXscore was able to predict the primary endpoint of cardiac death, non-fatal MI and late revascularization, independently of age, sex, presence of diabetes and index revascularization. There was a 6% increased risk in having a MACE for each additional point in the score. Patients with SXscore_HIGH_ had a significantly increased risk for all-cause, cardiovascular and cardiac death.

Previous studies have shown the ability of the SXscore to predict MACE in different scenarios. LEADERS,^9,10^ SIRTAX^11^ and RESOLUTE^8^ studies included patients with acute coronary syndromes as well as patients submitted to elective PCI. MI-SYNTAXscore Study,^12^ STRATEGY and MULTISTRATEGY^13^ studies were done in patients with acute MI. The ACUITY^7^ trial included patients with acute coronary syndromes. Finally, Serruys et al. summarized the results of five studies, analyzing data from 6.508 patients, with the same results.^14^ Differently from our research, all of those studies included only patients submitted to percutaneous revascularization procedures, and none of them focused on patients with suspected or stable CAD. The group of patients without significant CAD, although many had CAD with diameter stenosis lesser than 50%, served as the reference category to compare outcomes with those of patients in the SXscore_LOW-INTERMEDIATE_ and SXscore_HIGH_ categories. We understand that our proposal is different from the application originally proposed for the score, in which patients without lesions treatable with surgery or percutaneously are excluded, but it is in line with the objectives of the study.

The comparison of the SXscore performance with the traditional CAD anatomical scores was not explored in our investigation. The presence of positive SXscores (44.6%) was similar to the frequency of patients with significant CAD (47%) detected by quantitative angiographic analysis done in a proportion of the patients of our cohort.^21^ Traditional angiographic scores have also predicted the incidence of MACE in previous studies,^2^ but those scores do not take into account difficulties in performing myocardial revascularization. Despite their prognostic ability, they have not been incorporated to clinical practice, where the number of diseased vessels has been used to estimate the anatomical severity of disease. In this cohort, patients with one-vessel and multivessel disease had a risk for MACE approximately similar to the SXscore low-intermediate and high, respectively. Nonetheless, almost half of the patients with multivessel disease were classified in the SXscore_LOW-INTERMEDIATE_ category.

Based on the results of the SYNTAX trial,^5^ in which patients with low SYNTAX scores had similar outcomes regardless of the type of revascularization, our findings have a clinical implication - patients who would otherwise be referred to CABG could also be revascularized percutaneously. Considering that visual characterization of multivessel disease leads to referring patients to surgical revascularization, calculation of the SXscore could better stratify patients who would indeed benefit from this procedure (SXscore_HIGH_ category).

In this study, patients underwent an elective procedure and, as a result, there was a large proportion of patients with no significant CAD, who might also have lesions below 50%. Those patients did not fulfill the criteria for a positive score and served as a comparison group. Therefore, patients were classified into two categories: 1 to 22, and equal to 23 or greater, which correspond to the later defined categories of low-intermediate (0-22) and high (23 or higher).^6^ The proportion of patients classified as SXscore_LOW-INTERMEDIATE_ and submitted to percutaneous revascularization was higher than patients submitted to surgical revascularization, which reflects the current clinical practice and complies with the findings of the 5-year follow-up of the randomized clinical SYNTAX trial.^6^ At the time the study was conducted, drug-eluting stents were not available for use in the Brazilian public health system. In addition, current evidence indicates surgical revascularization for patients with high SXscores.^6^ Patients with high surgical risk who were not deemed eligible for surgical revascularization by surgeons received percutaneous treatment. There was an unexpected finding of 3.4% of patients without significant CAD who were submitted to PCI. Patients with non-obstructive CAD represent a large proportion of patients undergoing coronary angiography. Subjective evaluation of coronary anatomy associated with the clinical and noninvasive information might have influenced the decision-making process and could explain this finding.

Our study has some limitations and strengths that should be addressed. We restricted our analysis to anatomical criteria, considering neither left ventricular function nor myocardial ischemia and viability. Nonetheless, our patients did not have clinically unstable disease or classes III or IV heart failure, and the anatomical criteria frequently prevail to reach a therapeutic decision. In addition, a recent post-hoc analysis of the COURAGE trial has demonstrated that anatomical criteria and not ischemia burden were able to predict cardiovascular events.^22^ Although most follow-up procedures have been conducted in our hospital, different types of stents were implanted, which could affect the likelihood of stent thrombosis or reinterventions.^23^ However, analysis including only MI and cardiac death did not change the estimates. Exclusion of patients submitted to valve replacement did not change the results either. Another limitation is the number of events, which accounted for the large confidence intervals. Despite having investigated almost 1,000 patients, more than 50% did not have significant CAD, which reflects real life practice of a tertiary center performing diagnostic angiographies. Studies with larger sample size and conducted at other centers are necessary to confirm our findings and their external validity. The high inter-observer reproducibility of the examiners is among the strengths of our investigation, similar to some^24^ but different from other studies.^25,26^ This performance could be explained by the extensive training in assessing SXscore done by the interventional cardiologists and the fact that both underwent training at the same hospital.

### Clinical Implications

In clinical practice, the number of epicardial vessels with more than 50% stenosis is used to assess prognostic information, and angiographic scores are rarely used. Recently, the use of scores has been shown to improve the standardization of clinical decision-making. For instance, the EUROSCORE^27^ or the STS score^28^ are routinely used in the decision making process for the indication of CABG.^29^ For the management of multivessel CAD, current guidelines formally recommend the use of the SXscore as well as the EUROSCORE.^30^ Our data expand the indications of the SXscore for the prognostic evaluation of patients referred for diagnostic angiography.

## Conclusion

In conclusion, in patients with suspected CAD submitted to elective coronary angiography, SXscore independently predicts MACE. Its routine use in this setting could identify patients with worse prognosis.
